# Evaluation of graded recession of inferior oblique muscle for correction of different grades of V-pattern strabismus

**DOI:** 10.1186/s12886-023-03210-x

**Published:** 2023-11-16

**Authors:** Shaimaa Hady Sokeer, Ahmed L. Ali, El-Sayed S. Arafa, Amr M. Awara, Heba M. Shafik

**Affiliations:** Department of Ophthalmology, Faculty of Medicine, Tanta University, Tanta, Egypt

**Keywords:** V pattern strabismus, Inferior oblique overaction, Graded recession, Dissociated vertical deviation

## Abstract

**Background:**

V pattern identification is essential for proper strabismus management. Graded recession is a tailored approach to treat inferior oblique overaction (IOOA). The aim is to evaluate the efficacy of graded recession of inferior oblique muscle for correction of different grades of V pattern.

**Methods:**

Forty patients from 3 to 18 years old with V pattern strabismus and primary IOOA were evaluated by prism cover test to assess the grade of IOOA and amplitude of V-pattern. Graded recession of IO muscle depends on the amplitude of the V-pattern and degree of IOOA. Eight mm recession for amplitude 15 PD to 20 PD and mild IOOA (10 PD-15 PD or + 1) ,10 mm recession for amplitude 20–30 PD and moderate IOOA (15–25 PD or + 2) and maximum recession for amplitude more than 30 PD and marked IOOA (≥ 25 PD or + 3). Simultaneous correction of the horizontal deviation was performed. Follow up after I week,1 month ,3 month and 6-month. Trial Registration Number (TRN) (NCT05786053) on 23/3/2023.

**Results:**

The mean age of the study patients was 9 ± 4.261. Twenty patients (50%) had V-pattern esotropia, 12 (30%) exotropia, 4 (10%) orthotropic and four (10%) had Dissociated vertical deviation (DVD). Four cases 10% were of grade 1, 20 cases (50%) grade 2 and 16 cases (40%) were of grade 3. Of eighty eyes, 66 eyes (82.5%) were fully corrected with no residual IOOA, and 14 eyes (17.5%) were under corrected. V-pattern was corrected in 28 cases 70% and only 12cases (30%) had residual V-pattern grade 1.

**Conclusions:**

Graded recession is an effective procedure for correction of V pattern strabismus with various grades of primary inferior oblique overaction. It can be tailored according to the the degree of IO overaction which is significantly related to the grade of V pattern. The 8 mm recession for IO was significantly related to recurrence or inadequate break of the V pattern in our studied cases. The grade of IOOA correlates with the amplitude of V-pattern. The amount of recession was planned according to preoperative IOOA and grade of V-pattern with frequent undercorrections obtained by the standard 8 mm recession. A + 2 overaction merits a 10-mm recession of the inferior oblique. A + 3 or + 4 overaction merits a 14-mm maximal recession.

## Background

V-pattern horizontal deviations are the most common of alphabetic pattern strabismus [1].

While the only effective treatment is surgery, routine surgery, without specifically addressing the pattern, often fails, and special surgical procedures need to be used. V pattern is clinically significant when there is a difference between upgaze and downgaze of more than 15 prism diopters (PD) [2].

Diagnosis of the V-pattern is achieved by prism cover and alternate cover testing to assess the angle of deviation in different fields of gaze, especially those in primary gaze, upgaze, and downgaze. Fixation on a distant target, while the proper refractive correction is being worn, allows recording of accurate measurements [3]. Nearly 12-50% of patients with horizontal strabismus express vertical incomitance or significant pattern [1]. The concept of pattern in strabismus was first introduced by Duane in 1897.He illustrated the “V” pattern in bilateral superior oblique palsy [4].

Oblique muscle dysfunction is recently considered the most accepted theory explaining the etiology of pattern strabismus. Grades of V-pattern are dependent on the difference between the angle of deviation in both upgaze and downgaze. Grade 1 from 10to 15 PD, grade 2 from 15 to 25 PD, and grade 3 more than 25 PD [5].

Inferior oblique muscle overaction (IOOA) is extremely common with horizontal strabismus. About 70% of esotropes and 30% of exotropes express IOOA. IOOA was proposed to be the main etiology of V-incomitance and is sometimes associated with dissociated vertical deviation (DVD). IOOA is classified into primary and secondary. The primary type is usually bilateral, with unclear etiology, but the secondary type is unilateral and is caused by either ipsilateral superior oblique (SO) palsy or contralateral superior rectus palsy [6, 7]. Clinically, the primary type includes hypertropia of the adducted eye, minimal vertical deviation in the primary position, and minimal head tilt, and Bielschowsky test is negative, on the contrary, the secondary type includes pronounced vertical deviation in the primary position, hypertropia of the paralyzed adducted eye, head tilt is marked, and Bielschowsky test is characteristically positive [3, 8].

If V incomitance exceeds 15 PD, inferior oblique surgery is indicated in the presence of IOOA. There are many surgical procedures for weakening of IO muscle like graded recession, anteriorization, Z-myotomy, myectomy, disinsertion, and denervation [9]. The purpose of the study was to evaluate the efficacy of graded recession of IO muscle for correction of different grades of V -pattern.

## Patients and methods

This prospective randomized study of forty patients with primary inferior oblique over action with V pattern of more than 15 PD was done in Tanta University Eye Hospital, Egypt from October 2020 to January 2022. Retrograde Clinical Trial registration for the surgical techniques used was obtained (NCT05786053) on 23/3/2023. The first posted date is 27/3/2023.

A detailed orthoptic assessment was carried out. Examination was done for chin up/down, head tilt, nystagmus, and A, V, or X pattern. Horizontal and vertical angles of deviation were measured using a prism cover test (PCT) [10]. PCT was done with full correction spectacle worn at 6 m in primary position, chin up, chin down, right, left, and near fixation. Findings of PCT were described in prism diopters, which were converted into approximate degrees by taking half of its value. Versions were also checked for overaction of inferior oblique [11]. It was also differentiated from trochlear palsy where vertical deviation (VD) is incomitant. Park 3-step test was done in routine to isolate paretic muscle in case of vertical deviation [11]. Recently, IOOA has been graded according to the degree of hypertropia of the adducted eye. A scale ranging from + 1 to + 4 overelevation has been used. In the maximal lateral version, + 1 IOOA for hypertropia of approximately 10–15 PD, + 2 for (15–25) PD, + 3 for (25–35) PD, and more than 35 PD was + 4 [12]. Stereopsis was assessed using Tetmus fly test. It is a screening test for gross stereopsis (400–700 s per arc). Although It is a near stereoacuity test, it was used for gross screening of stereoacuity as most of study patients were children. Grades of v-pattern are dependent on the difference between the angle of deviation in around 15–20 PD in up and down gaze. Grade 1 from 15 to 20-degree, grade 2 from 20 to 30 degrees, and grade 3 more than 30 degrees.

The study included all the patients who were clinically diagnosed as V incomitance of more than 15 PD due to Primary IOOA associated with either exotropia (Exo), esotropia (Eso), hypertropia, or DVD. Patients with poor fixation and Significant neurodevelopmental delay were excluded from the study. Three grades of IO recession were performed. Grading depends on two factors, the amplitude of the V-pattern and degree of IOOA.

These two factors are interdependent i.e., the more sursoadduction presents the more amplitude of the V-pattern and vice versa.

The three grades of recession were 8 mm recession for amplitude 15 PD to 20 PD and mild inferior oblique overaction (within 10–15 PD or + 1),10 mm recession for amplitude 20 PD to 30 PD degrees, and moderate inferior oblique overaction (between 15 PD and 25 PD or + 2), maximum recession for amplitude more than 30 PD and marked inferior oblique overaction (more than 25 PD or + 3,+4).In IO maximal recession (14 mm), we attach the anterior portion of IO to a point about 2 mm at the lateral side of the insertion of the inferior rectus muscle (without J shaped attachment). Maximal recession and anteriorization (AT) surgery was reserved for V incomitance associated with DVD. In these cases, IO was attached 2 mm at the lateral border of the inferior rectus muscle with (J) shaped attachment. In cases showing asymmetric IOOA in both eyes, different recession procedures were performed according to the grade in each eye. Co-existing horizontal strabismus either Exotropia or Esotropia were simultaneously corrected. The decision was taken according to measurements in the primary position to perform the appropriate horizontal muscle surgery. Motor alignment was considered Successful when orthotropic or ≤ 10 prism diopter (Δ) exotropic or esotropic at 6 m. Also, V incomitance was considered surgically successful when measurement difference between up and downgaze is < 15PD with refractive correction worn [8]. Inferior oblique surgery was considered successful when IOOA was eliminated postoperatively during follow-up visits.

The primary outcome measures were the postoperative distant angle measurements in primary position and upgaze. The secondary outcome measures were the absence of inferior oblique overaction and the break of V pattern. Informed written consent to perform surgery was obtained.

Informed written consent was obtained from all subjects and/or their legal guardian(s) **to publish** identifying information/images in an online open-access publication.

Follow-up was performed at 1 month,3 months, and 6 months after surgical correction.

### Statistical analysis

We used the Statistical Package for Social Sciences version 23.0 for Windows (SPSS Inc., Chicago, IL) to perform statistical analyses. Quantitative data were expressed as mean ± standard deviation (SD). Qualitative data were expressed as frequency and percentage. A comparison of continuous variables for the mean angle of deviation before and after surgical correction was achieved using a paired t-test.

Comparison of proportions between two qualitative parameters was achieved using Chi-square (X2) test. A *p*-value of ≤ 0.05 was considered significant.

**Table 1:** Criteria of patients with V-pattern strabismus with inferior oblique overaction in our study (range-mean-SD)

		Minimum	Maximum	Range	Mean	SD
Age of Onset		1.00	9.00	8.00	2.80	1.71
Age at the time of surgery		3.00	18.00	15.00	9.00	4.26
SE*	RT eye	-6.30	9.00	15.30	1.58	3.25
	LT eye	-3.50	9.50	13.00	1.81	2.71
BCVA**	RT eye (log MAR)	0.10	1.00	0.90	0.67	0.36
	LT eye (log MAR)	0.30	1.00	0.70	0.78	0.29
Pretreatment Angle of Deviation (PD)		-90.00	100.00	190.00	11.00	45.81

SE* stands for spherical equivalent

BCVA** stands for best corrected visual acuity. **PD** (prism diopters)

**Table 2:** Correlation between different grades of IOOA and grades of V-pattern in the study patients

Grade of IOOA		Grade of V Pattern			Total
		1	2	3	
0		2	4	0	6
		2.50%	5.00%	0.00%	7.50%
1	N	0	12	4	16
	%	0.00%	15.00%	5.00%	20.00%
2	N	4	12	10	26
	%	5.00%	15.00%	12.50%	32.50%
3	N	2	8	6	16
	%	2.50%	10.00%	7.50%	20.00%
4	N	0	4	12	16
	%	0.00%	5.00%	15.00%	20.00%
Total	N	8	40	32	80
	%	10.00%	50.00%	40.00%	100.00%
Pearson’s R	**X** ^**2**^	**3.388**			
	***P*** **-value**	0.001*			

**Table 3:** Post-operative success/unsuccess of Graded recession of Inferior Oblique muscle in the study patients (80 eyes)

Pre-operative grade IOOA	Post-operative IOOA		
	Success	Unsuccess	Total
0	2	4	6
+ 1	8	8	16
+ 2	24	2	26
+ 3	16	0	16
+ 4	16	0	16
Total	66(82.5%)	14	80
Chi-square	**X** ^**2**^	**30.272**	
	***P*** **-value**	0.001*	

**Success** = no residual IOOA **Unsuccess** = IOOA

## Results

This study included 40 patients with V-pattern strabismus, 20 of them had V-pattern esotropia, 12 of them had V- pattern exotropia, 4 with hypertropia, and four cases with DVD.

**Fig. 1:**
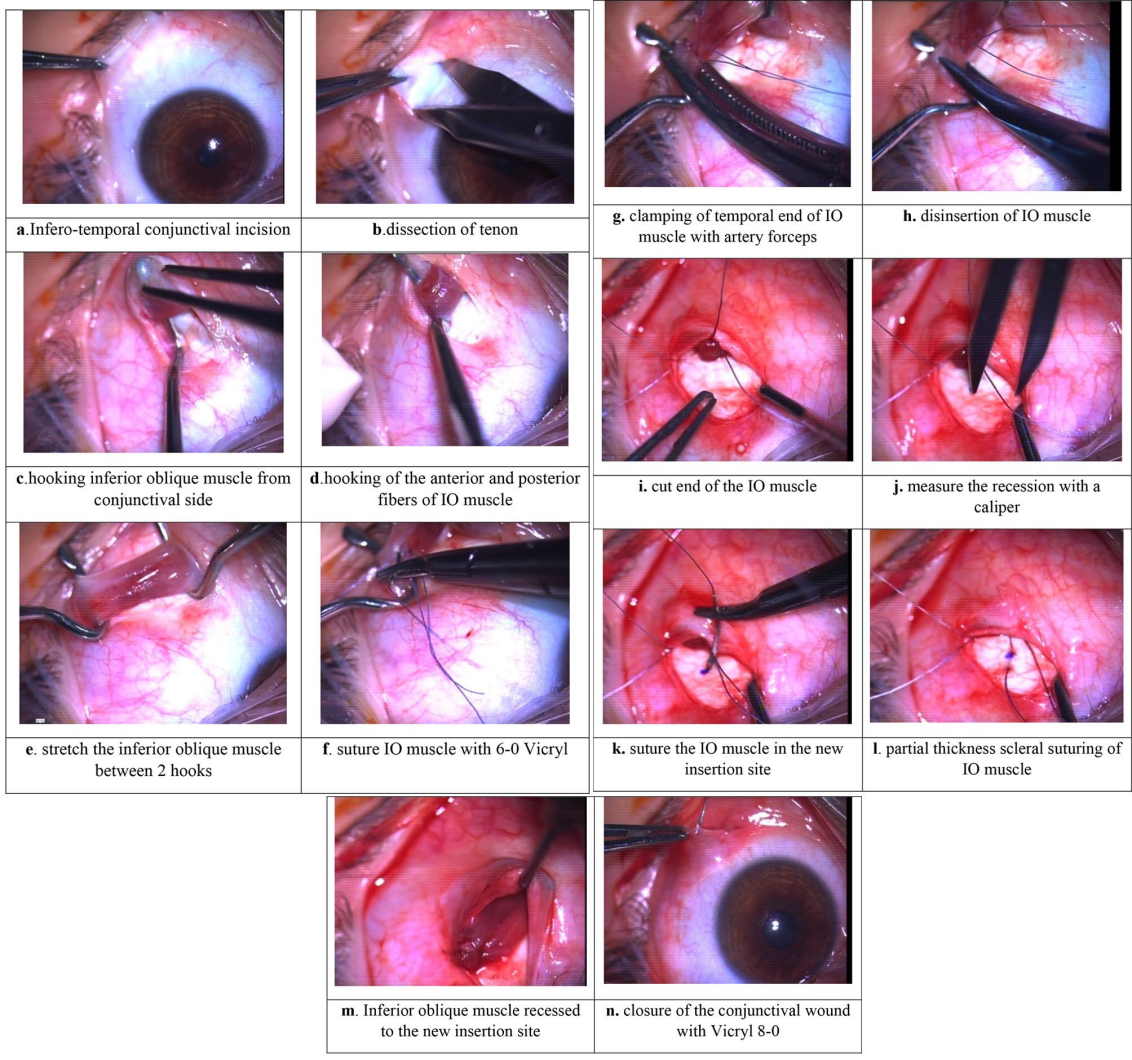
Surgical steps of graded recession of inferior oblique muscle

**Fig. 2:**
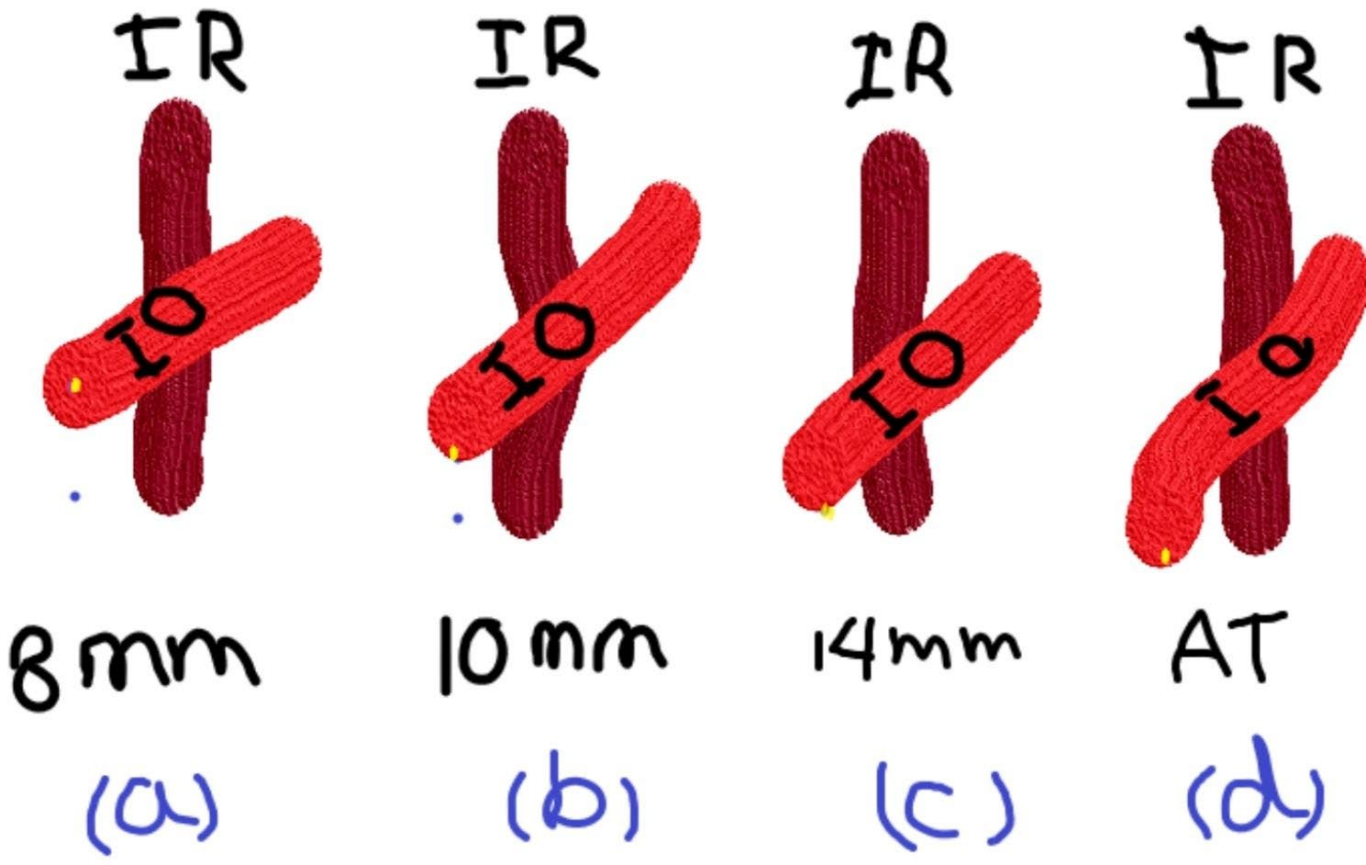
Inferior oblique placement according to IOOA (graded recession). **(a)** 8-mm recession (appoint 2 mm lateral and 4 mm posterior to IR muscle). **(b)** 10-mm recession (appoint 2 mm lateral and 2 mm posterior to IR muscle). **(c)** maximal IO recession(14 mm) (appoint 2 mm lateral to IR muscle). **(d)** maximal recession and anteriorization of IO (AT) (appoint 2 mm lateral to IR muscle with J- deformity). Note that yellow point refers to site of IO attachment measured by surgical caliper

The mean age of the study patients was 9 years old with a range between (3–18) years as shown in Table (1). There was a significant correlation between different grades of IOOA and grades of V-pattern in the study patients (*P*-value was 0.001*) as shown in Table (2). Twenty-four patients (60%) were below 10 years, 16 patients (40%) were above 10 years, 18 patients (45%) were males, and 22 patients (55%) were females. As regards the grade of V-pattern, four cases (10%) were of grade 1, 20 cases (50%) were of grade 2, and 16 cases (40%) were of grade 3. Regarding IOOA, the study included 40 patients (80 eyes). Six eyes had no inferior oblique muscle overaction (IOOA) and had no I.O muscle surgery,16 eyes were of grade 1 IOOA and had 8 mm recession, 26 eyes were of grade 2 IOOA had 10 mm recession, 16 eyes were of grade 3 IOOA and had the maximum recession of IO muscle, sixteen eyes were of grade 4 IOOA,14 of them had a maximum recession with anteriorization and two eyes underwent maximum recession. Surgical steps are shown in (Fig. 1). Inferior oblique placement point in GR is demonstrated in (Fig. 2). Regarding post-operative results of IO surgery, (66) eyes of (80) were fully corrected and (14) eyes (17.5%) showed under correction with success rate (82.5%) as shown in Table (3). Of six eyes that did not show IOOA initially, four eyes had IOOA later. They underwent IO surgery later to address IOOA. However, the second surgical intervention wasn’t included in the statistical analysis of this study. Regarding post-operative success in the correction of V- pattern, 1 month after surgery, (12) cases (30%), were corrected, three months after surgery (26) cases (65%) were corrected and six months after surgery (28) cases (70%) were corrected regarding V-pattern as shown in Tables (4, 5, 6, 7). There was a significant correlation between the pre- and post-operative state of stereopsis (P value is 0.001*) as shown in Table (8). Of four cases with DVD who underwent maximal recession and anteriorization with J shaped attachment, one case had anti-elevation syndrome with limited elevation in all directions. There was mild improvement of duction limitation gradually over 6 months.

**Table 4:** Correlation between the grade of Inferior oblique muscle recession and post-operative amplitude of V-Pattern. (ONE month after surgery)

1 Month Post- operative V Pattern Grade		Grade of IO Recession					Total
		None	8 mm	10 mm	Maximum	Maximum Recession anteriorization	
**0**	**N**	0	0	4	8	0	12
	**%**	0.0%	0.0%	10%	20%	0.0%	30%
**1**	**N**	2	4	12	4	4	26
	**%**	5%	10%	30%	10%	10%	55%
**2**	**N**	0	0	0	0	2	2
	**%**	0.0%	0.0%	0.0%	0.0%	5%	5%
**Total**	**N**	2	4	16	12	6	40
	**%**	5%	10%	40%	30%	15%	100.0%
**Chi-square**	**X** ^**2**^	**23.675**					
	***P*** **-value**	0.003*					

**Table 5:** Correlation between the grade of Inferior oblique muscle recession and post-operative amplitude of V-Pattern. (Three months after surgery)

3 Months Postoperative Grade of V Pattern		Grade of IO Recession					Total
		None	8 mm	10 mm	Maximum	Maximum Recession anteriorization	
**0**	**N**	2	2	10	10	2	26
	**%**	5%	5%	25%	25%	5%	65%
**1**	**N**	0	2	6	2	4	14
	**%**	0.0%	5%	15%	5%	10%	35%
**Total**	**N**	2	4	16	12	6	40
	**%**	5%	10%	40%	30%	15%	100.0%
**Chi-square**	**X** ^**2**^	**5.934**					
	***P*** **-value**	**0.204**					

**Table 6:** Correlation between the grade of Inferior oblique muscle recession and post-operative amplitude of V-Pattern. (Six months after surgery)

6 Months Post- operative Grade of V Pattern		Grade of IO Recession					Total
		None	8 mm	10 mm	Maximum	Maximum Recession ant	
**0**	**N**	2	2	12	10	2	28
	**%**	5%	5%	30%	25%	5%	70%
**1**	**N**	0	2	4	2	4	12
	**%**	0%	5%	10%	5%	10%	30%
**Total**	**N**	2	4	16	12	6	40
	**%**	5%	10%	40%	30%	15%	100.0%
**Chi-square**	**X** ^**2**^	**6.667**					
	***P*** **-value**	**0.155**					

**Table 7:** Post-operative success in the correction of V- pattern in study patients

	Success		Unsuccess	
	N	%	N	%
1 month	12	30	28	70
3 months	26	65	14	35
6 months	28	70	12	30

**Table 8:** Post-operative stereopsis in study patients above 6 years(30 patients) in relation to the pre-operative state

State of Stereopsis	Degree of stereopsis as No. and percentage of patients			Median stereo acuity (seconds per arc)
	Nil	Moderate	Bifixation	
**Pre-operative**	4(13.3%)	14(46.7%)	12(40%)	600(15-nil)
**6-month post-operative**	4(13.3%)	6(20%)	20(66.7%)	700(15-nil)
**Chi-square**	**X** ^**2**^	**37.714**		
	***P*** **-value**	0.001*		

## Discussion

For weakening of IOOA, surgery should either diminish the muscle tension (myectomy and recession) or change the mechanical function vector. The vector of mechanical function is changed by moving the insertion site of IO muscle. To decrease IO muscle tension and change functional insertion, IO recession with anteriorization is done. Cases with Severe IOOA (grade + 3 or + 4) or associated with DVD are treated with anteriorization of IO to the inferior rectus insertion. If significant IOOA is associated with horizontal deviation, simultaneous IO weakening procedure should be addressed with horizontal muscle surgery. Keeping in mind that IO weakening surgery has no significant effect on the horizontal deviation in the primary position. Clinically, IO weakening surgery is indicated for IOOA of + 2 or more. If IOOA is bilateral yet asymmetric, inferior oblique weakening surgery should be performed on both eyes even when one eye expresses minimal or only + 1 IOOA. This should be done to avoid unmasking the minimal IOOA [13–15].

The outcomes of IO weakening techniques were compared in different studies [16–19]. Cases with V-pattern caused by (IOOA)were included in Apt and Call study who adopted graded recession (GR) of the inferior oblique (IO). They got a successful outcome by this procedure which has become widely used for similar cases. This was the first step toward anterior transposition (AT) [20]. In his study, Parks has compared different IO weakening procedures. He studied patients with bilateral IOOA without hypertropia and compared IO recession, myectomy between origin and inferior rectus, and myectomy at insertion and disinsertion. Park found that recession was the most effective procedure for correction of IOOA [21]. The best advantage of this procedure is that it allows titration of the weakening procedure according to the grade of IOOA.

Surgical success was seen in 66 of 80 eyes (82.5%). A successful outcome was defined as no hypertropia in adduction at 6 months postoperatively. Fourteen eyes (17.5%) showed under correction. Of six eyes that did not show IOOA initially, four eyes had IOOA later 6 months postoperatively. Eight eyes (50% of grade + 1) with grade + 1 IOOA was under-corrected by the 8-mm recession. Undercorrections were frequently obtained by the standard 8 mm recession. Some cases that had unilateral inferior oblique maximal recession and or anteriorization elicited IOOA of the other side later.

Inferior oblique muscle was recessed to a point 2 mm lateral and 3 mm posterior to inferior rectus muscle insertion in patients with + 1 or + 2 IOOA by Ozosy Ercan. He had surgical success in 92 of 95 eyes (96.8%). IOOA persisted in 3 eyes (3.1%). One patient had + 2 IOOA and 2 had + 1 IOOA [22, 23]. Parks concluded that the IO recession procedure was superior. The inferior oblique muscle was recessed 10 mm for + 1 or + 2 IOOA, and 12 mm for + 3 overaction. For + 4 IOOA,14 mm recession was done which is the maximum recession. Parks has also reported that 15% of cases had IOOA recurrence with the IO recession surgery, in comparison with 79% with myectomy at the origin, 53% with disinsertion, and 37% with hyperfunction recurrence with myectomy at the insertion [21]. In general, post-operative short-term follow-up wasn’t of great value after IO muscle surgery because IOOA can recur insidiously over 2 years. Therefore, to accurately evaluate IO weakening procedure, long-term follow-up is mandatory. Recently, a 25% recurrence rate of IOOA after surgery was reported by Wilson and Parks. They followed up on the cases for 3 years. However, repeat IO surgery was performed in only 6% of patients [15]. Our results of the frequency of residual overaction were higher than Park and Ercan et al. Many reports in the literature state that under corrections are frequently obtained by the standard 8 mm recession. Elliott and Nankin, therefore, introduced the maximum recession technique to overcome this problem [16]. Undercorrection was observed in this study by 8 mm recession of IO. However, significant correction of IOOA was observed by 10 mm and maximal recession.

We had 4 cases of V-pattern associated with DVD, 2 cases (50%) were of grade 2 V-pattern and the other two cases were of grade 3. Inferior oblique maximal recession with anteriorization reduced a presurgical vertical imbalance. Full correction was observed in (50%) of the patients and under correction in 2 cases (50%). In their study of 37 eyes in 22 patients Guemes & Wright, AT was done. They proved the efficacy of AT procedure to normalize the version and to correct the hypertropia in the primary position of gaze [23]. However, AT should be reserved for cases with moderate to severe IOOA as Ziffer et al. explained as Anterior transposition may sometimes cause limitation of upgaze. [24] Of four cases with V-pattern associated with DVD, one had limited elevation in all directions postoperatively with mild improvement during follow-up visits. In our study, we had no inferior oblique underaction of more than − 2 postoperatively. Bacal & Nelson had no reported complications with this procedure in 55 patients [25].

In our study, 30% of the patients (12 cases) had a residual V pattern of grade 1 associated with under-correction of IOOA at the final follow-up visit. Complete normalization of the IOOA was seen in 66 of 80 eyes postoperatively with two patients who had a postoperative limited elevation of grade 1.

There was an increase in the effect on the reduction of vertical deviation (VD) in adduction from day one postoperatively to 6 months postoperatively in study patients. This coincides with the findings of Metten et al., who could find an increase in the operative effect on reduction of the VD (with and without anterior transposition). During the follow-up period, we were mainly concerned with recording the correction of V- incomitance, rather than residual IOOA. Vertical incomitance significantly improved 6 months after surgery.

Reduction of the IOOA or V incomitance with statistically significant results was observed by Polati et al. In 45.5% (20 eyes) the IOOA has returned to normal. However, a recorded reduction of IOOA was observed in 34.1% (15 eyes), but with persistent + 1 IOOA. “Satisfactory” correction of the V incomitance, defined as less than 10 PD residual incomitance was recorded in 77.3% of the cases [26]. The conventional recession resulted in a mean reduction in V-pattern as reported by Kamlesh et al. by about 26.9 PD (25.83 PD in V-exotropia and 19.75 PD in V-esotropia).On the other hand, significant correction in V-exotropes and V-esotropes was reported by Taha et al [27]. An average correction was observed by Burian et al. and Prakash et al. [28, 29]. A correction of 1.25–11.25 PD was also reported by Ziffer in an arbitrary scale of 0 and + 4 for IO hyperfunction [26]. Park’s 10 mm recession resulted in an improvement of 12.76 ± 3.3 PD, Elliot and Nankin’s anteropositioning resulted in an improvement of 18.80 ± 6.5 PD, and 12.76 ± 3.3 PD by pure anteropositioning as reported by Prakash, et al. However, their studies included patients of both primary and secondary IOOA; so we cannot directly compare our results and theirs [29].

## Conclusion

Graded recession of IO is an effective procedure for correction of V pattern strabismus with various grades of primary inferior oblique overaction. It can be tailored according to the degree of IO overaction which is significantly related to the grade of V pattern. The 8 mm recession for IO was significantly related to recurrence or inadequate break of the V pattern in our studied cases.

## Data Availability

The data sets used during the current study are available from the corresponding author on reasonable request.
